# Prevention of Pulmonary and Venous Thromboembolism Post Coronary Artery Bypass Graft Surgery – Literature Review

**DOI:** 10.21470/1678-9741-2018-0345

**Published:** 2020

**Authors:** Mansour Jannati, Alireza Abdi Ardecani

**Affiliations:** 1 Department of Cardiovascular Surgery, Faghihi Hospital, Shiraz University of Medical Sciences, Shiraz, Iran.

**Keywords:** Coronary Artery Bypass, Venous Thromboembolism, Comorbidity, Risk Factors, Postoperative Period

## Abstract

**Objective:**

The current review evaluates recent literature on the different aspects of prophylaxis in postoperative pulmonary and venous thromboembolism and their main risk factors.

**Methods:**

The literature survey was carried out based on the PubMed data using the keywords “coronary artery bypass graft” and “venous thromboembolism” as components of the search field title.

**Results:**

Studies reported several risk factors for postoperative thromboembolism including advanced age, postoperative immobilization, type of thromboprophylaxis, obesity, and location of the surgery.

**Conclusion:**

According to the studies, tailored prophylaxis could be easily adapted to decrease the intensity and duration of postoperative thromboembolism in a patient with several disorders and comorbidities, especially in cardiovascular disease.

**Table:** 

Abbreviations, acronyms & symbols				
ACCP	= American College of Chest Physicians		MI	= Myocardial infarction
ACS	= Acute coronary syndrome		MRI	= Magnetic resonance imaging
AF	= Atrial fibrillation		NOACs	= Non-vitamin K antagonists oral anticoagulants
CABG	= Coronary artery bypass graft		PAD	= Peripheral arterial disease
CAD	= Coronary artery disease		PE	= Pulmonary embolism
CDC	= Centers for Disease Control and Prevention		PESI	= Pulmonary embolism severity index
CHD	= Coronary heart disease		RCT	= Randomized controlled trials
CUS	= Compression ultrasonography		RR	= Relative risk
CVD	= Cardiovascular disease		RV	= Right ventricular
DVT	= Deep vein thrombosis		sPESI	= Simplified pulmonary embolism severity index
HFS	= Hip fracture surgery		THA	= Total hip arthroplasty
HT	= Hypertension		TIA	= Transient ischemic attack
IL	= Interleukin		TKA	= Total knee arthroplasty
LMWH	= Low-molecular-weight heparin		TTE	= Transthoracic echocardiography
MCP	= Monocyte chemotactic protein		UFH	= Unfractionated heparin
MDCT	= Multi-detector computed tomographic		VKAs	= Vitamin K antagonists
METHRO	= Melagatran for Thrombin Inhibition in Orthopaedic Surgery		VTE	= Venous thromboembolism

## INTRODUCTION

Cardiovascular disease (CVD) consists of a variety of disorders affecting the vascular structure, including hypertension (HT), atherosclerosis, coagulopathies, coronary heart disease (CHD), myocardial infarction (MI), stroke, and venous thromboembolism (VTE)^[1]^. VTE comprises two related disorders, including deep vein thrombosis (DVT) and pulmonary embolism (PE)^[2]^, and it is considered as the third most common disease among life-threatening disorders, such as MI and stroke^[3]^.

Several manifestations have been determined as the most frequent risk factors of VTE, including old age, previous VTE, chronic heart failure, MI, malignancy, thrombophilia, prolonged immobility, hip fracture, the existence of central catheters, estrogen treatment, major surgery, and trauma^[3]^.

It has been also shown that among all clinical predictors of VTE, old age, chronic heart failure, MI, major surgery, and trauma are more associated with PE, whereas DVT is mainly related to malignancy and thrombophilia^[3]^. Due to the difficulty of PE diagnosis and treatment, the mortality rate of PE is higher than of other CVDs, such as MI^[4]^.

In a global survey performed in 2015, it has been shown that there was a relative lack of public awareness about thrombosis overall, and especially about the symptoms and signs of DVT and PE. It also applies to estrogen-containing medications as a risk factor for VTE^[5]^. Evidence showed that the prevalence of VTE significantly increases after spine surgery in patients with walking disability before operation, elastic stocking, HT, lumbar surgery, and diabetes^[6]^.

A full dose of anticoagulant therapies, including unfractionated heparin (UFH), UFH and low-molecular-weight heparin (LMWH) as parenteral anticoagulants, fondaparinux, orally active vitamin K antagonists (VKAs) and non-vitamin K antagonists oral anticoagulants (NOACs), or thrombolysis, is recommended for both acute and long-lasting VTE patients^[7,8]^. One of the promising drugs for prevention of recurrent unprovoked VTE is Aspirin, which has low cost, with a once-daily application without dose monitoring^[9]^. Further preclinical research is required to determine the role of different risk factors and mechanisms in thrombosis formation to develop novel anti-inflammatory treatments, reducing the incidence of VTE in postsurgical patients.

In this review, we aimed to define DVT as the most common cause of PE, the risk factors of VTE and its therapeutic strategies, the VTE and atherothrombosis relationship, the prophylaxis for VTE, and, moreover, to evaluate the efficacy of these prophylactic strategies in reducing VTE without increasing the risk of post-cardiac surgery complications.

### Data Collection

The literature survey was carried out based on the PubMed data using the keywords “coronary artery bypass graft (CABG)” and “venous thromboembolism” as components of the search field title. We found 95 articles, among which those that included the objectives of the search were selected. Articles in languages other than English, texts that were not complete articles, and those published before 1990 were excluded.

VTE in Cardiac Surgery and CABG

Cardiac surgeries are accompanied with many risk factors for DVT development, such as general anesthesia, long hospital stay, long duration of surgery, too many manipulations in vascular structures throughout the surgery, immobilization, and etc^[10]^. Risk factors for DVT in CABG patients include obesity, cardiac failure, advanced age, female gender, hyperlipidemia, smoking, pregnancy, and etc^[11]^. Subsequent to cardiac surgery, DVT may cause important complications^[6]^. VTE and PE may lead to mortality following cardiac surgery, and they are the fifth most common reason of readmission to hospital after CABG^[12]^. However, in most patients, DVT remains undetected and its signs normally become apparent within a few weeks after surgery^[11]^. The incidence of PE following cardiac surgery is 0.5-3.9%^[11]^, and the incidence of detection of DVT is 13% of cases who underwent cardiac surgery^[13]^.

### Common Approaches in PE Diagnosis

Diagnostic tests including chest X-ray and electrocardiogram are applied to detect PE in suspected patients^[14-17]^. Based on different clinical predisposing factors, various scales have been used to predict PE probability, including PE severity index (PESI) and simplified PESI (sPESI)^[18]^. But the application of D-dimer for PE confirmation has been reduced due to its high negative predictive value. Among these, imaging tests such as invasive pulmonary angiography theoretically are widely used as a gold standard for a timely diagnosis of PE. But the specificity and sensibility of the multi-detector computed tomographic (MDCT) angiography are more suitable for clinical practice^[18,19]^. The alternative for MDCT is the pulmonary perfusion/ventilator scintigraphy. The application of magnetic resonance imaging (MRI)-angiography is rare in clinical practice because of its low sensitivity and high inconclusive results in PE diagnosis^[20]^. One of the best methods in PE diagnosis, particularly at patient’s bed, is the noninvasive transthoracic echocardiography (TTE), which has low cost and provides respectable information on the degree of pulmonary HT and the severity of right ventricular (RV) dysfunction^[18]^. In patients with hemodynamic instability, echocardiography has been applied as a valuable test, in which appears the RV dysfunction leading to immediate PE treatment and survival^[19,20]^.

### DVT as the Most Common Cause of PE

DVT is considered as the origin of PE in a lower limb in most cases. It has been recommended to apply sequential noninvasive surveys for DVT in patients with suspected PE. Venous ultrasonography is mostly used for diagnosis of patients with proximal and symptomatic DVT than with asymptomatic distal and recurrent DVT^[21]^.

Among different standards which have been recommended for DVT diagnosis, the lack of vein compressibility occurred by venous thrombosis and evaluated by compression ultrasonography (CUS) is considered as a common criterion with a 90% sensitivity and 95% specificity for symptomatic DVT^[22,23]^. Detection of a proximal DVT in PE suspected patients is the main sign of anticoagulant therapy without doing additional tests^[20]^.

### Surgery and Postsurgical Inflammation as Risk Factors of VTE and its Therapeutic Strategies

It has been shown that surgery leads to an increase of the risk of VTE, including DVT and PE. However, morbidity and mortality rates of postsurgical VTE remain high all over the world, even with the contemporary therapeutic strategies, such as mechanical manipulation and pharmacological prophylaxis^[24]^.

A series of triad factors, known as Virchow’s triad, have contributed to form venous thrombi, including endothelial dysfunction, low blood flow, and a hypercoagulable state when they occur at the same time^[25,26]^. Low activity or stasis in patients after surgery may cause venous thrombosis formation^[25,26]^. Another factor involved in thrombosis is inflammation, which leads to a hypercoagulable state and endothelial damage. Initiation of the inflammatory response has been attributed to the pro-inflammatory cytokines rising, also called cytokine “storm”, after surgery, which provides a pro-thrombotic environment followed by several cellular processes, such as neutrophil extracellular traps formation, platelet activation, the tissue factor-bearing micro-particles localization, and endothelial injury. In a research on the incidence of symptomatic VTE by type of surgery, White et al. found out that the percentage of incidence/case was high in orthopaedic (1.2%) and vascular (1.1%) surgeries^[27]^.

In a literature review of studies on the relevance of predisposing factors involved in VTE after total hip arthroplasty (THA)^[28]^, several factors have been introduced, including the diagnosis of hip fracture^[29]^, malignancy^[30]^, particularly with chemotherapy, antiphospholipid syndrome^[31]^, immobility or reduced mobility^[32]^, history of VTE^[33]^, tamoxifen^[34]^ and raloxifene^[35]^ therapy, estrogen replacement therapy, stroke^[36]^, atherosclerosis^[37]^, old age^[38]^, and obesity^[28,39]^. Other factors, such as diabetes mellitus^[40]^, certain cardiovascular conditions^[30]^, leukemia^[41]^, varicose veins, and smoking, presented low risk^[28]^. Findings of a systematic review and meta-analysis of different observational studies and randomized controlled trials (RCT) showed that some factors, such as previous history of VTE, obesity, left or right ventricular failure, prolonged bed rest, mechanical ventilation, or a central venous catheter application, are considered as common risk factors of VTE^[42]^.

Based on the 2012 American College of Chest Physicians (ACCP) guidelines on VTE prevention in orthopaedic surgery patients, the use of VTE prophylaxis, such as Aspirin, have been recommended in patients undergoing major orthopaedic surgeries, including THA, total knee arthroplasty (TKA), and hip fracture surgery (HFS)^[43]^. Prolonged low-dose Aspirin therapy has also been recommended by the ACCP guidelines for secondary prevention of coronary artery disease (CAD) events^[44,45]^. In a review by Cohen et al.^[46]^, Aspirin has been introduced as the key component in the management of CVD events, including secondary prevention of CAD and acute coronary syndrome (ACS), and non-cardioembolic stroke or transient ischemic attack (TIA), primary prevention of stroke in patients with low thrombotic risk of atrial fibrillation (AF), and primary and secondary prevention of peripheral arterial disease (PAD). In a study performed by Hawkins, the role of oral direct thrombin inhibitors in the VTE prevention was evaluated^[47]^. Ximelagatran, a new low-molecular-weight oral prodrug, has been introduced as the direct thrombin inhibitor melagatran of VTE in patients with orthopaedic surgery compared to warfarin or fondaparinux^[47]^. The safety and efficacy of melagatran and its prodrug ximelagatran in VTE prophylaxis have been established in several European trials of the Melagatran for Thrombin Inhibition in Orthopaedic Surgery (METHRO)^[48,49]^.

### What is the Association Between VTE and Atherothrombosis?

It has been shown that VTE and atherothrombosis display the same pathophysiology of inflammation, hypercoagulability, endothelial injury, and cardiovascular risk factors, including obesity, HT, diabetes mellitus, cigarette smoking, hypercholesterolemia, congestive heart failure, and metabolic syndrome^[4]^. Steffen et al.^[50]^ found out that there is a significant relationship between metabolic syndrome and risk of VTE in a survey of 20,374 patients.

One of the best-considered components in the VTE epidemiology is that pan-cardiovascular syndromes, including CAD, PAD, and CVD, are associated with VTE manifestation. Evidence evaluated that several medical conditions, such as immobility, heart failure, chronic lung disease, and chronic kidney disease, increase VTE risk in patients with symptomatic atherosclerotic CAD.

### Inflammation Markers and Risk Factors Involved in VTE

Several markers have been attributed to VTE pathogenesis, including interleukin (IL)-6, IL-8, and monocyte chemotactic protein (MCP)-1^[51]^. VTE risk factors were divided to inherited thrombophilias, including VTE in many family members, idiopathic or recurrent VTE, recurrent spontaneous abortions, factor V Leiden, prothrombin gene mutation 20210, deficiencies of protein C, protein S, or antithrombin, and acquired risk factors, including old age, cancer, immobility, and recent trauma, surgery, or hospitalization^[4,52]^. Among different cancers, patients with stomach and pancreatic cancers are at high risks in developing VTE^[53]^. Other cancers, including lung, gynecologic, bladder, and testicular cancers, and lymphoma develop VTE with high incidence^[53]^. Middle-aged women are prone to develop VTE during the first 12 postoperative weeks^[54]^. Pregnancy, oral contraceptives containing estrogen, and hormone replacement are considered as important VTE risk factors in women^[55]^.

### VTE Management in Pregnancy

It has been recommended that the same diagnostic and therapeutic strategy should be applied to pregnant patients^[56]^. The first step of diagnosis is real-time or duplex ultrasound, especially in iliofemoral DVTs, and then ultrasound or X-ray venogram is repeated^[56]^. In VTE suspected patients, a ventilation-perfusion lung scan is recommended. UFH or LMWH are applied for treatment, which should be maintained during the pregnancy and should be altered to warfarin postpartum for at least six weeks^[57]^. Another controlling approach in pregnant patients with VTE is a screening of thrombophilia^[56]^.

### The Role of Prophylaxis in VTE Reduction Without Increasing the Risk of Complications After Cardiac Surgery

One of the most important risk factors for VTE is hospitalization. Based on reports of the Centers for Disease Control and Prevention (CDC), most hospitalizations are complicated by VTE, including DVT and PE, in patients with age ≥ 18 years annually. In a systematic review and meta-analysis in 2015, data showed that VTE prophylaxis was significantly correlated with a reduced risk of PE (with relative risk [RR], 0.45; 95% confidence interval) or symptomatic VTE (RR, 0.44) compared to the control group^[42]^. Any sign of bleeding or cardiac tamponade requiring reoperation due to pharmacological VTE prophylaxis without systemic anticoagulation therapy was not detected in patients undergoing cardiac surgery^[42]^.

## DISCUSSION

For surgical procedures including cardiac surgeries, especially post CABG, that are not correlated with any comorbidities, such as appendectomy and cholecystectomy, or with coexisting comorbidities, advancing age will be attributed as a significant cause and predictor of thromboembolism.

In a study by Ambrosetti et al.^[58]^, a relatively high rate of DVT after CABG was shown (about 17%). In their study, two of the 47 patients with DVT acquired PE, being fatal in one patient. Goldhaber et al.^[59]^ reported an approximately 22% DVT rate after CABG. A study by Reis et al.^[60]^ revealed that the incidence of DVT after cardiac bypass surgery was 48.3%, diagnosed at about 6.5 days after surgery. Viana et al.^[64]^ observed isolated DVT in 4% and simultaneous DVT in 8% of CABG patients.

Studies showed that PE occurred in 0.4 to 9.5% of patients after bypass surgery, and it was fatal in 0.3 to 1.7% of the cases^[61,62]^. Beck KS et al. showed that the PE rate after CABG was 6.2% and it mostly occurred at the segmental or subsegmental level^[63]^. Viana et al.^[64]^ observed isolated PE in 13% and simultaneous PE in 8% of CABG patients. In Goldhaber et al.^[59]^ study, the frequency of PE was 0.6% in patients with bypass and the rate of fatal massive PE was 0.3%. In another study by Josa et al.^[62]^, the frequency of PE was 3.2% after cardiac surgery.

It is expected that the VTE incidence strongly associated with age remains and will be increased after cardiac surgery. Previous studies have reported that advancing age is related to postsurgical VTE^[65,66]^. Although VTE contributed as a main preventable factor of morbidity and mortality in hospitalized patients, controversy still exists on the efficacy and safety of VTE prophylaxis after cardiac surgery. Further research, in preclinical models, is required to determine the precise association of VTE prophylaxis and management of thrombosis. This may simplify the improvement of novel anti-inflammatory managements to decrease VTE occurrence in the postsurgical settings. In this regard, it is essential that physicians be aware of the pro-inflammatory mechanisms involved in thrombus formation in their patients.

The findings of this study are mainly based on the evidence available in previous and recent review studies^[67,68]^. With these evidences, we found out that VTE prophylaxis could reduce PE and symptomatic VTE risk in post-cardiac surgery without any bleeding and cardiac tamponade in patients.

Ho et al.^[42]^ reported that initiating pharmacological VTE prophylaxis soon after cardiac surgery for patients who have no active bleeding is highly recommended. Close et al.^[69]^ recommended that all patients after cardiac surgery initiate the use of heparin prophylaxis the day after their surgery and continue this up to discharge. Sarker et al.^[70]^ reported that combined treatment with rivaroxaban and heparin is effective in a case of post-CABG DVT patient.

In a study by Kulik et al.^[11]^ on the effectiveness of preventative therapy for VTE after CABG, they found out that the administration of chemical or mechanical preventative therapies to CABG patients does not appreciably lower the risk of VTE.

In line with a systematic review and meta-analysis study, some packages of evaluated VTE prophylaxis had sufficient safety and efficacy in PE and symptomatic VTE reduction^[42]^. However, they concluded that sufficient data did not exist regarding the efficacy of one type of VTE prophylaxis compared to other types or if a specific therapeutic agent was superior to the others^[42]^. In terms of VTE in hip arthroplasty, most conducted studies and their findings indicated that platelets play an important role in the VTE pathogenesis^[71]^. In a survey of 13,356 patients with hip fracture or undergoing hip arthroplasty, the efficacy of antiplatelet agents on first episode and recurrence of VTE was found^[72]^. In this regard, Aspirin has been recommended by different researchers as one of the most common drugs to prevent VTE after surgery, including CABG. On the other hand, Aspirin resistance is a common phenomenon observed in high doses during the first week after cardiac surgery^[73]^. A low dose of antiplatelet agents, such as Aspirin, has not been recommended by surgeons due to its weak function in VTE prevention in high-risk cardiac patients^[73]^. Application of Aspirin alternative, such as UFH or LMWH, offering additional VTE protection has not shown an increase in risks of bleeding, pericardial effusion, and cardiac tamponade^[74]^. In a cohort study, Kulik et al.^[75]^ reported that application of UFH or LMWH during 48 hours in post-CABG patients were not associated with bleeding risk compared to no VTE prophylaxis. A large quantity of evidence showed that bleeding could occur after overdose of anticoagulation therapy^[67]^.

Findings of the current study showed that there was an evidence gap regarding VTE and PE prevention, especially after CABG, suggesting that it is essential to perform more RCT and cohort studies to evaluate the precise efficacy and safety of various anticoagulant agents in reducing VTE and their cost-effectiveness after cardiac surgery. In conclusion, the development of various therapeutic strategies and prophylaxis is needed to prevent PE and symptomatic VTE events after cardiac surgery.

**Table:** 

Authors' roles & responsibilities	
MJ	Substantial contributions to the conception or design of the work; or the acquisition, analysis, or interpretation of data for the work; drafting the work or revising it critically for important intellectual content; final approval of the version to be published
AAA	Substantial contributions to the conception or design of the work; or the acquisition, analysis, or interpretation of data for the work; drafting the work or revising it critically for important intellectual content; final approval of the version to be published
